# Tumor Mutation Burden Forecasts Outcome in Ovarian Cancer with BRCA1 or BRCA2 Mutations

**DOI:** 10.1371/journal.pone.0080023

**Published:** 2013-11-12

**Authors:** Nicolai Juul Birkbak, Bose Kochupurakkal, Jose M. G. Izarzugaza, Aron C. Eklund, Yang Li, Joyce Liu, Zoltan Szallasi, Ursula A. Matulonis, Andrea L. Richardson, J. Dirk Iglehart, Zhigang C. Wang

**Affiliations:** 1 Department of Cancer Biology, Dana-Farber Cancer Institute, Boston, Massachusetts, United States of America; 2 Center for Biological Sequence Analysis, Technical University of Denmark, Lyngby, Denmark; 3 Department of Medical Oncology, Dana-Farber Cancer Institute, Boston, Massachusetts, United States of America; 4 Children's Hospital Informatics Program at the Harvard-Massachusetts Institutes of Technology Division of Health Sciences and Technology (CHIP@HST), Harvard Medical School, Boston, Massachusetts, United States of America; 5 Department of Pathology, Brigham and Women’s Hospital, Boston, Massachusetts, United States of America; 6 Department of Surgery, Brigham and Women’s Hospital, Boston, Massachusetts, United States of America; University of Hawaii Cancer Center, United States of America

## Abstract

**Background:**

Increased number of single nucleotide substitutions is seen in breast and ovarian cancer genomes carrying disease-associated mutations in *BRCA1* or *BRCA2*. The significance of these genome-wide mutations is unknown. We hypothesize genome-wide mutation burden mirrors deficiencies in DNA repair and is associated with treatment outcome in ovarian cancer.

**Methods and Results:**

The total number of synonymous and non-synonymous exome mutations (Nmut), and the presence of germline or somatic mutation in *BRCA1* or *BRCA2* (mBRCA) were extracted from whole-exome sequences of high-grade serous ovarian cancers from The Cancer Genome Atlas (TCGA). Cox regression and Kaplan-Meier methods were used to correlate Nmut with chemotherapy response and outcome. Higher Nmut correlated with a better response to chemotherapy after surgery. In patients with mBRCA-associated cancer, low Nmut was associated with shorter progression-free survival (PFS) and overall survival (OS), independent of other prognostic factors in multivariate analysis. Patients with mBRCA-associated cancers and a high Nmut had remarkably favorable PFS and OS. The association with survival was similar in cancers with either *BRCA1* or *BRCA2* mutations. In cancers with wild-type BRCA, tumor Nmut was associated with treatment response in patients with no residual disease after surgery.

**Conclusions:**

Tumor Nmut was associated with treatment response and with both PFS and OS in patients with high-grade serous ovarian cancer carrying *BRCA1* or *BRCA2* mutations. In the TCGA cohort, low Nmut predicted resistance to chemotherapy, and for shorter PFS and OS, while high Nmut forecasts a remarkably favorable outcome in mBRCA-associated ovarian cancer. Our observations suggest that the total mutation burden coupled with *BRCA1* or *BRCA2* mutations in ovarian cancer is a genomic marker of prognosis and predictor of treatment response. This marker may reflect the degree of deficiency in BRCA-mediated pathways, or the extent of compensation for the deficiency by alternative mechanisms.

## Introduction

 Reliable biomarkers predicting resistance or sensitivity to anti-cancer therapy facilitate selection of proper therapeutic drugs in individual cancer patients. In breast cancer, the estrogen receptor and HER2 (erbB-2/neu) are used clinically to make therapeutic decisions about endocrine therapy and HER2-targeted drugs, respectively [1,2]. Both the estrogen receptor and HER2 participate in pathways that promote cancer growth. Likewise, *BRCA1* and *BRCA2* participate in error-free repair of double-strand DNA breaks by homologous recombination (HR) and inherited mutations in these genes predispose to breast and ovarian cancers [3]. Ovarian cancers carrying *BRCA1* and *BRCA2* mutations (mBRCA) display massive chromosomal alterations [4,5], and are more sensitive to DNA cross-linking agents containing platinum, and to PARP inhibitors [6,7]. Patients with high-grade serous ovarian cancer who carry germline mBRCA experience a longer progression-free survival (PFS) and better overall survival (OS) than non-carriers [6,8,9]. Therefore, *BRCA1* and *BRCA2* may be considered biomarkers that predict response to platinum-containing chemotherapy and to PARP inhibitors. However, in previous studies 15-18 % of BRCA-associated ovarian cancers responded poorly to platinum-based chemotherapy regimens, and either recurred or progressed shortly after initial surgery and chemotherapy [8,9]. 

Most sporadic high-grade serous ovarian cancer and triple-negative breast cancer do not have mutations in BRCA genes, but a subset of these tumors do exhibit massive chromosomal aberrations and responsiveness to DNA damaging chemotherapy [9-11]. An appealing hypothesis posits chromosomal aberrations are a gauge of the degree of impairment in HR. Proposed surrogates for HR defects include measures of chromosomal aberrations including whole genome loss of heterozygosity (LOH) and telomeric allelic imbalance [11,12]. Lack of Rad51 foci after DNA damage may also mark cells with impaired HR [13]. 

Recently, a significantly higher mutation burden was detected by whole genome or exome sequencing in breast and ovarian cancer with mBRCA, compared with their counterparts carrying the wild-type *BRCA1* and *BRCA2* (wtBRCA) genes [14,15]. Whole exome sequencing of high-grade serous ovarian cancers was reported by The Cancer Genome Atlas (TCGA) consortium[9]. The DNA sequence of ovarian cancers was compared to germline DNA sequence from the same subject to make somatic mutation calls. Identified mutations included base substitutions, insertions or deletions [9,15]. The vast majority of mutations were single base substitutions [9]. Accumulation of genome-wide mutations may be the consequence of unique mutational processes associated with DNA repair deficiency in tumors carrying *BRCA1* or *BRCA2* mutations. 

Since ovarian cancers with mutations in *BRCA1* or *BRCA2* are more sensitive to platinum-containing chemotherapy, we asked whether the total number of somatic mutations in ovarian cancer predicts sensitivity to chemotherapy and clinical outcome. We used whole exome sequencing data from TCGA to enumerate somatic mutations and compared this to chemotherapy sensitivity, progression free survival (PFS) and overall survival (OS). A significant association between the total number of somatic exome mutations per genome (Nmut) and patient outcomes was observed in patients whose ovarian cancers possessed mutations in *BRCA1* and *BRCA2*. 

## Results

### Association of mutation burden with chemotherapy sensitivity and outcome

**Figure 1:**
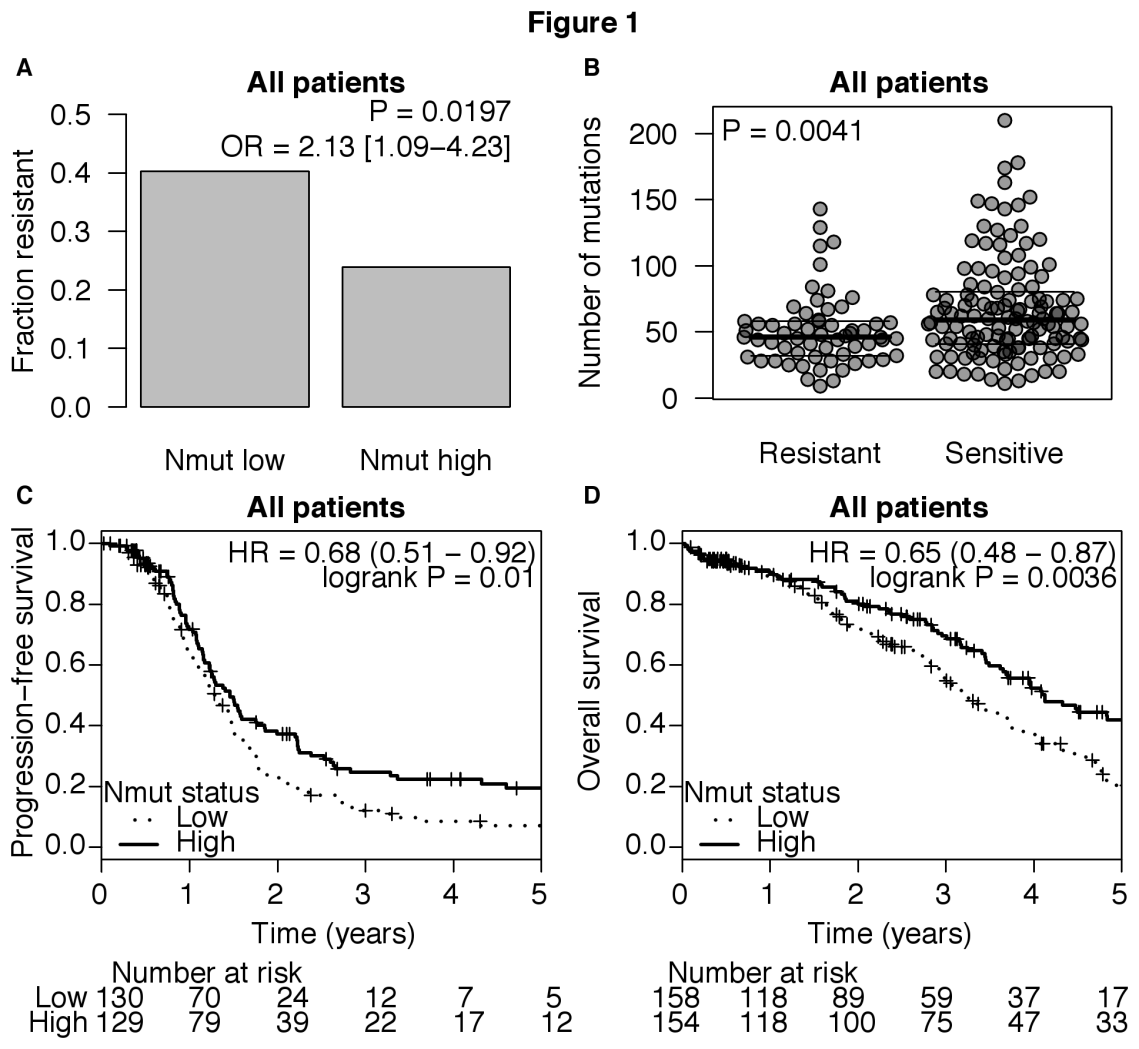
Total number of exome mutations (Nmut) and clinical outcome in high-grade serous ovarian cancer. All patients received platinum and most received taxanes in combination. **A**) Tumors were separated into Nmut high and low groups defined by the median Nmut across the whole cohort and compared to the rate of chemotherapy resistance. The significance of the differences was determined by Fisher’s exact test. **B**) The number of mutations (Nmut) for each tumor was compared in chemotherapy resistant and sensitive patients and is shown by dot plots. Median and 25-75 percentiles are indicated by horizontal lines. P-value is derived from the Wilcoxon rank-sum test. **C**) Kaplan-Meier analysis compared the progression-free survival (PFS) and **D**) overall survival (OS) between patients with high and low tumor Nmut. Patients that were progression-free or still alive at the time of last follow-up were censored (+). Numbers of patients at risk at each interval are given below the graphs. P-values are obtained by Log-rank test.

 Using data from TCGA, we found that 95% of mutations in exomes of ovarian cancer are single base substitutions. Across the TCGA cohort of 316 tumors, the number of exome mutations in individual cancers (Nmut) varies widely, from 9 to 210 (median 54.5, Table S1). To determine whether Nmut is associated with chemotherapy resistance after initial surgery, we separated patients into Nmut high and low groups based on the median Nmut of the whole cohort. A higher rate of resistance to initial chemotherapy was observed in Nmut low compared to the Nmut high group (40.2 vs. 23.9 %, Figure 1A). Nmut was lower in treatment-resistant patients than sensitive patients (median 46 vs. 59, Figure 1B). Cox regression showed a correlation between Nmut and progression-free survival (PFS) or overall survival (OS) (*P* = 0.013 and 0.0014, respectively, Table 1). Kaplan-Meier analysis showed a significantly longer PFS and OS in the Nmut high group compared to the Nmut low group (Figure 1C and 1D). 

**Table 1:** Univariate and Multivariate analysis of Nmut and other clinical variables with PFS and OS.

		**Univariate**				**Multivariate**		
		**HR^a^**	**95% CI^b^**	**P^c^**		**HR**	**95% CI**	**P**
**All cases**								
Nmut^d^	**PFS**	**0.944**	**(0.990-0.999)**	**0.013**		**0.955**	**(0.991-1.000)**	**0.042**
	**OS**	**0.926**	**(0.988-0.997)**	**0.0014**		**0.913**	**(0.986-0.996)**	**0.0001**
Stage	**PFS**	**1.466**	**(1.072-2.005)**	**0.017**		1.38	(0.985-1.935)	0.061
	OS	1.325	(0.960-1.828)	0.087		1.221	(0.865-1.724)	0.256
Residual^e^	**PFS**	**1.183**	**(1.026-1.365)**	**0.021**		1.158	(0.999-1.342)	0.052
	**OS**	**1.267**	**(1.091-1.470)**	**0.0019**		**1.245**	**(1.065-1.455)**	**0.006**
Age (yrs)	PFS	0.995	(0.982-1.009)	0.492		0.998	(0.984-1.013)	0.828
	**OS**	**1.019**	**(1.005-1.033)**	**0.0075**		**1.025**	**(1.010-1.040)**	**0.001**
**Mbrca**								
Nmut	**PFS**	**0.817**	**(0.968-0.993)**	**0.002**		**0.856**	**(0.971-0.998)**	**0.027**
	**OS**	**0.828**	**(0.967-0.996)**	**0.011**		**0.821**	**(0.966-0.995)**	**0.0082**
Stage	PFS	1.694	(0.745-3.853)	0.209		1.415	(0.600-3.338)	0.428
	OS	1.304	(0.539-3.154)	0.555		1.1	(0.396-3.055)	0.856
Residual	PFS	0.999	(0.723-1.379)	0.993		0.979	(0.695-1.379)	0.904
	OS	1.362	(0.959-1.936)	0.084		1.389	(0.961-2.009)	0.081
Age (yrs)	PFS	0.987	(0.960-1.016)	0.378		0.999	(0.967-1.031)	0.928
	OS	1.017	(0.985-1.049)	0.301		1.023	(0.990-1.058)	0.175
**wtBRCA**								
Nmut	PFS	0.987	(0.994-1.003)	0.593		0.989	(0.994-1.004)	0.648
	OS	0.966	(0.992-1.001)	0.159		**0.948**	**(0.990-1.000)**	**0.032**
Stage	PFS	1.369	(0.980-1.913)	0.065		1.234	(0.859-1.772)	0.255
	OS	1.224	(0.871-1.719)	0.244		1.119	(0.778-1.608)	0.545
Residual	**PFS**	**1.231**	**(1.048-1.447)**	**0.011**		**1.219**	**(1.030-1.443)**	**0.021**
	**OS**	**1.195**	**(1.011-1.414)**	**0.037**		1.192	(1.000-1.421)	0.051
Age (yrs)	PFS	0.994	(0.979-1.010)	0.466		0.998	(0.982-1.015)	0.841
	**OS**	**1.017**	**(1.001-1.033)**	**0.035**		**1.024**	**(1.007-1.041)**	**0.0051**

a Hazard ratio

b 95% confidence interval

c P-value from Cox proportional hazard regression

d HR for Nmut is expressed the ratio per 10 mutations

e Residual disease left after initial surgery

### Effect of BRCA1 and BRCA2 on mutation burden and outcome

**Figure 2:**
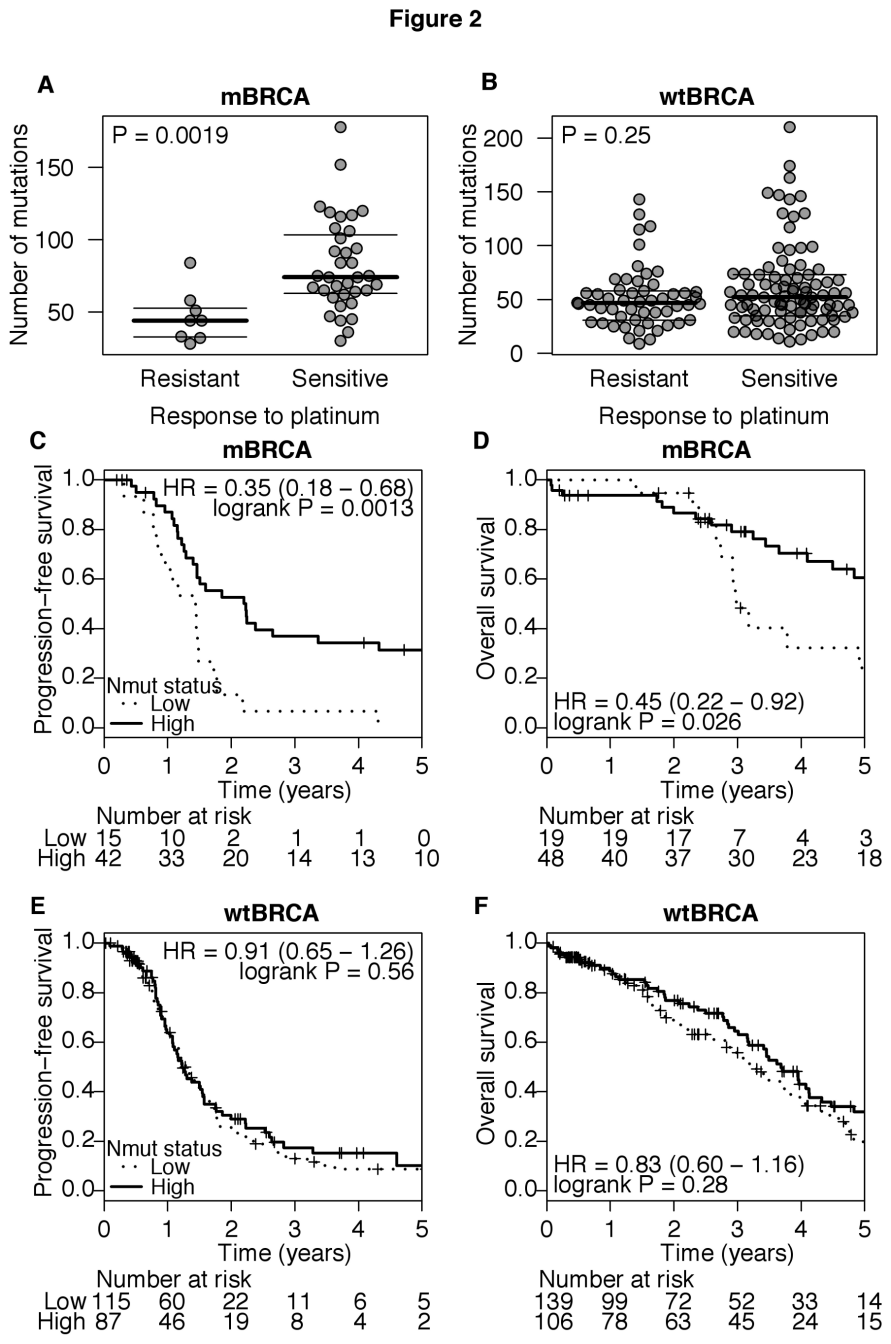
Total number of exome mutations (Nmut) and clinical outcome in high-grade serous ovarian cancer with germline or somatic mutations in *BRCA1* or *BRCA2* (mBRCA) or with wild-type *BRCA1* and *BRCA2* (wtBRCA). **A**) Nmut in tumors with mBRCA. Chemotherapy resistant and sensitive ovarian cancers are shown by dot plots. P-value is derived from the Wilcoxon rank-sum test. **B**) Nmut in tumors with wtBRCA. Chemotherapy resistant and sensitive tumors are shown with dot plots of each tumor as in Figure 1. Median and 25-75 percentiles are indicated by horizontal lines. P-value is derived from Wilcoxon rank-sum test. **C**) Kaplan-Meier analysis compared PFS and **D**) OS between patients with high and low Nmut in their mBRCA-associated tumors. **E**) Kaplan-Meier analysis compared PFS and **F**) OS in patients with high and low Nmut in their wtBRCA tumors. The median for Nmut was computed from the whole cohort of 316 tumors. In Kaplan-Meier analyses, patients that were progression-free or still alive at the time of last follow-up were censored (+). Numbers of patients at risk at each interval are given below the graphs. P-values are obtained from Log-rank test.

 Seventy patients either carried a germline *BRCA1* or *BRCA2* mutation or possessed tumors bearing somatic *BRCA1* or *BRCA2* mutations (mBRCA). We found no differences in tumor Nmut, PFS or OS between patients with germline and tumor somatic mutations in *BRCA1* and *BRCA2* (Figure S1). However, mBRCA-associated tumors possessed a higher Nmut than tumors without BRCA mutations (wtBRCA; median 67.5 vs. 49.5, Figure S2A). We separately analyzed the subset of patients bearing mBRCA and those with wtBRCA tumors, and compared tumor Nmut between chemotherapy resistant and sensitive patients. A higher tumor Nmut predicted a higher rate of response to chemotherapy after surgery in patients with mBRCA-associated tumors, but not in those with tumors that possessed only wtBRCA (Figures S2B and S2C). When we investigated all patients with tumors containing mBRCA, we found a significantly higher tumor Nmut in the treatment-sensitive group versus the treatment-resistant group (median 74 vs. 44, Figure 2A). In patients with wtBRCA tumors, there were no significant differences in Nmut between the treatment sensitive and resistant groups (median 52 vs. 47, Figure 2B). Cox regression showed a significant correlation between tumor Nmut and PFS and OS in patients with mBRCA-associated tumors (HR = 0.82, *P* = 0.002 and HR = 0.83, *P* = 0.011, respectively), but not in patients with wtBRCA tumors (Table 1). When patients with mBRCA-associated tumors were stratified by the median Nmut of the whole cohort, patients with high tumor Nmut showed a significantly longer PFS and OS (Figure 2C and 2D). PFS and OS in patients with mBRCA and low tumor Nmut were shorter, similar to patients with wtBRCA tumors (Figure 2C to 2F). In patients with wtBRCA tumors, there was no significant relationship between Nmut and PFS or OS (Figure 2E and 2F). Therefore, the effect of tumor Nmut on treatment response and outcome was chiefly confined to those tumors with either germline or somatic mutations in *BRCA1* or *BRCA2*. 

 In univariate and multivariate analysis, stage at presentation, size of residual tumors after debulking surgery, patient age and Nmut were associated with either PFS or OS in all patients with clinical follow-up (Table 1). Strikingly, for the patients with mBRCA-associated ovarian cancer, only Nmut was significantly associated with treatment outcome in both univariate and multivariate analysis. In multivariate analysis of cancers with wtBRCA, residual disease left after initial surgery was significantly associated with both PFS and OS. Nmut and age were significantly associated with OS, but not PFS in patients with wtBRCA (Table 1). These results show Nmut is significantly associated with clinical outcome and is independent of other prognostic factors in patients with mBRCA-associated tumors. 

 All 51 germline mutations in *BRCA1* and *BRCA2* were truncating mutations. Of the 21 somatic mutations in the two genes, 4 were missense and the others truncating. We examined location of the mutations in *BRCA1* and *BRCA2* genes for association with Nmut in tumors (Figures S3A and S3B). We separated BRCA mutations into ring, middle and BRCT domains of BRCA1 and N-terminal, RAD51 binding and C-terminal regions of BRCA2. Differences in Nmut among tumors with mutations in these regions of BRCA1 and BRCA2 were evaluated. No significant association was found between Nmut and mutations in different regions of BRCA1 or BRCA2 (Kruskal-Wallis test for multiple comparisons, *P* = 0.58 and *P* = 0.13, Figures S3C and S3D). 

**Figure 3:**
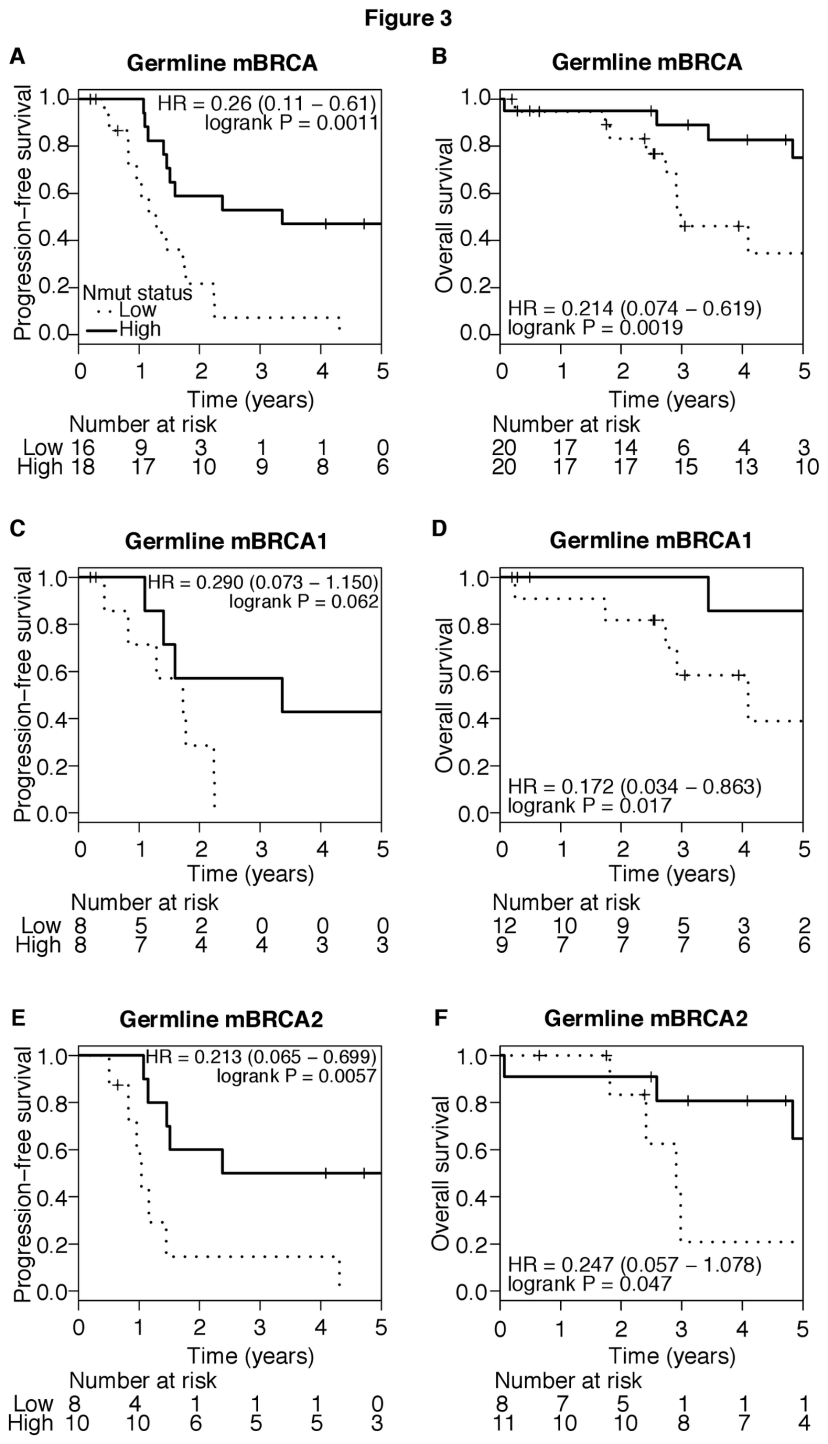
Tumor Nmut and clinical treatment outcome in ovarian cancer patients carrying BRCA germline mutations with LOH at the BRCA loci in tumors. **A**) Kaplan-Meier analysis compared PFS and **B**) OS between Nmut high and low ovarian cancers, all of which carried either a *BRCA1* or *BRCA2* germline mutation with LOH at the corresponding BRCA locus. **C** - **F**) Analysis in individual BRCA1 and BRCA2 mutation carrier groups. **C**) Kaplan-Meier analysis in patients with *BRCA1*-associated tumors comparing PFS, and **D**) OS. **E**) Kaplan-Meier analysis in patients with *BRCA2*-associated tumors comparing PFS, and **F**) OS. Nmut high and low are defined as a value above or below median Nmut of all mBRCA-associated tumors. Numbers of patients at risk at each interval are given below the graphs. *P*-values are calculated by log-rank test.

Fourteen mBRCA-associated tumors (6 somatic and 8 germline BRCA mutations) remained heterozygous at the mutated BRCA locus (Table S1). To avoid the influence of the wtBRCA allele, we tested for the association between tumor Nmut and clinical outcome in the subset of patients carrying BRCA germline mutations with LOH at the corresponding BRCA locus in their tumors. Cox regression revealed a significant correlation between Nmut and OS (HR = 0. 765, *P* = 0.021) and a trend toward significant correlation between Nmut and PFS (HR = 0. 837, *P* = 0.056). Kaplan-Meier analysis displays the remarkable differences in outcome between patients with high and low tumor mutation burden (Figure 3A and 3B). Despite small numbers, significant and consistent differences in PFS and OS were seen when *BRCA1* and *BRCA2* germline mutation carriers were evaluated separately (Figure 3C to 3F). These results support the conclusion that tumor Nmut is associated with both treatment response and clinical outcome within patients with inherited *BRCA1* or *BRCA2* mutations. 

We examined Nmut in tumors with known epigenetic changes in *BRCA1* (n = 31) and *RAD51C* (n = 8) in this TCGA dataset. Compared to tumors with wtBRCA and without methylation in the two genes, we observed a higher Nmut in tumors with *BRCA1* or *RAD51C* methylation, similar to tumors with mBRCA (Figure S4). The result suggests that epigenetic silencing in *BRCA1* and *RAD51C* may lead to accumulation of single base substitutions. However, in agreement with previously published results [9,15], the outcomes (PFS and OS) of patients with tumors harboring *BRCA1* methylation coupled with high Nmut were similar to patients whose tumors had low Nmut or wtBRCA1 (data not shown). The association between tumor Nmut and treatment outcome appears largely in cancers with *BRCA1* mutation, but not in those cancer with *BRCA1* epigenetic alteration.

### Correlation between Nmut and age or chromosomal damage

Nmut in tumors from patients with germline *BRCA1* or *BRCA2* mutations (BRCA mutations) increased with patient age at diagnosis (Figure S5A). However, this relationship was lost when tumors with somatic BRCA mutations were included or those with wtBRCA were analyzed separately, (Figures S5B and S5C). These finding are consistent with a distinct pathogenic process in germline BRCA-associated cancers with haplo-insufficiency of BRCA function in premalignant tissue, and those cancers that acquire BRCA mutations later in their development. A similar correlation between accumulated mutations and age was reported in cancers that arise from tissues which normally replicate during life (e.g., colonic epithelium), but are not seen in cancers from tissue normally dormant (e.g., cells in the exocrine pancreas)[16]. 

Both the fraction of LOH per genome (FLOH) and the number of episodes of telomeric allelic imbalance (NtAI) reflect the extent of tumor chromosomal damage [11,12]. Using TCGA SNP6 data from the same cohort, Nmut positively correlated with FLOH and NtAI in mBRCA-associated tumors; NtAI correlated with Nmut in wtBRCA tumors (Figure S6). The association between high mutation burden and high level of chromosomal damage suggests a link between the processes that produce or fail to repair these distinct types of DNA damage.

### Influence of residual disease on the association of mutation burden and outcome

**Figure 4:**
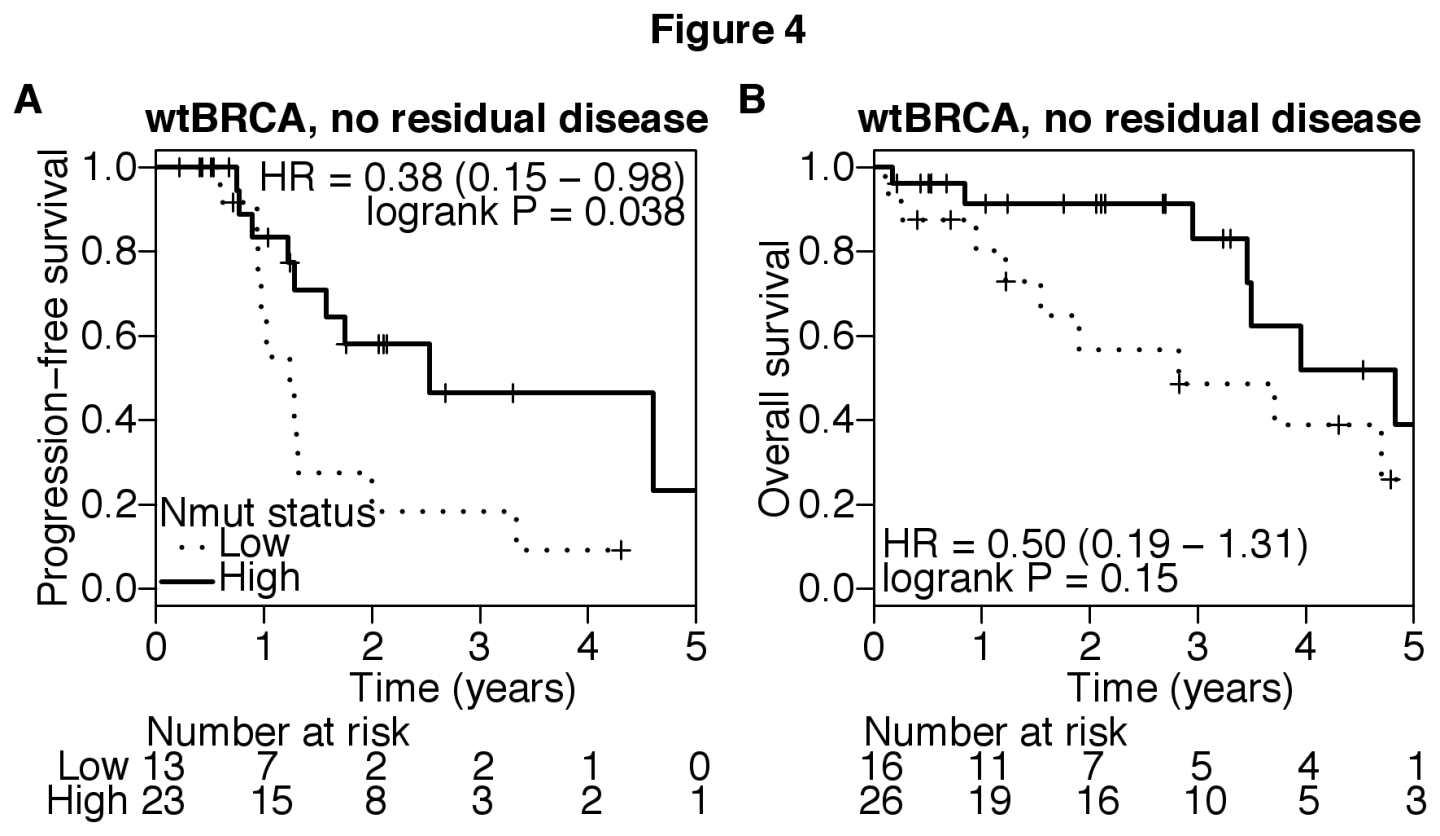
Nmut and treatment outcome in patients with wtBRCA tumors and no residual disease after initial surgery. Nmut high and low were defined by values above or below the median Nmut from all wtBRCA tumors in the cohort. **A**) Kaplan-Meier analysis compared PFS, and **B**) OS between tumor Nmut high and low in patients with wtBRCA tumors and no residual disease after debulking surgery. Numbers of patients at risk at each interval are given below the graphs. P-values are obtained from Log-rank test.

 Residual disease after initial surgery is a prognostic factor in ovarian cancer and was confirmed in both mBRCA- and wtBRCA-associated ovarian cancer (Figure S7). In patients with mBRCA-related cancers, those with a high tumor Nmut had better outcomes than those with a low tumor Nmut regardless of whether residual disease was present after initial surgery (Figure S8). Patients with no residual disease and a high tumor Nmut had an especially favorable outcome (5 year PFS was 58% and OS was 100%; Figure S8). In the subset of patients with wtBRCA tumors and no residual disease after surgery, high tumor Nmut predicted a longer PFS and a trend towards longer OS (Figure 4). No such differences were found in patients with wtBRCA tumors and residual disease after surgery (data not shown). Residual disease is a powerful prognostic factor, which may mask the effect of tumor Nmut in patients with wtBRCA tumors. The result suggests Nmut is potentially associated with treatment outcome in sporadic ovarian cancer with wtBRCA and no residual disease. 

## Discussion

 High-grade serous ovarian cancer in carriers of *BRCA1* or *BRCA2* has a better prognosis than the same disease in non-carriers, and may be more sensitive to cisplatin-based chemotherapy or to PARP inhibitors that target DNA repair [6-8]. However, within the group of women with somatic or inherited mutations in *BRCA1* or *BRCA2*, some patients will still have poor outcomes. There are currently no markers of treatment outcome in patients with mBRCA-associated ovarian cancer. Possible markers might include impaired apoptosis, multi-drug resistance and DNA repair proficiency. The present study sought to correlate whole-exome mutation burden in tumor tissue (Nmut) to treatment outcome in ovarian cancer patients, and to examine this relationship in patients with *BRCA1* and *BRCA2* mutations in their ovarian tumors. 

 The most remarkable association of Nmut with treatment response and outcome was seen within the subset of patients with mBRCA-associated tumors. A substantial proportion of patients with mBRCA-associated ovarian cancer but low Nmut experienced a relatively poor treatment outcome, and similar to patients with wtBRCA ovarian cancer. However, for women whose cancers were mBRCA-associated and had a high tumor Nmut, outcome was remarkably good. This was true for both *BRCA1* and *BRCA2* mutations, both germline and somatic mutations, and for tumors with LOH at the corresponding locus. In patients with mBRCA-associated cancers and no residual disease after initial surgery, those with high Nmut had especially good outcomes. In fact, long survival in high-grade serous ovarian cancer, when it is observed, may be attributable to mutation in either *BRCA1* or *BRCA2* when these genotypes are coupled with a high tumor Nmut. Nmut is a candidate genomic marker for predicting treatment outcome in patients with mBRCA-associated ovarian cancer. The association of Nmut and outcome may reflect the degree of deficiency in BRCA1- or BRCA2-mediated DNA repair pathway(s), or the result of compensation for the deficiency by alternative mechanisms. However, all of the patients in the TCGA cohort received platinum-based chemotherapy, and the beneficial effect of a BRCA1 or BRCA2 deficiency on OS may be due to improved treatment response, or due to the less lethal potential of mBRCA-associated cancers.

In our analysis of TCGA data, *BRCA1* mutation-associated ovarian cancer had a better outcome when coupled with a high tumor Nmut. In addition, *BRCA1* mutation-associated cancer that lost the wild-type *BRCA1* allele had a better outcome than ovarian cancer with only wild-type *BRCA1* (data not shown). It is unclear why *BRCA1* methylation, even coupled with high Nmut, does not translate into the same survival benefit seen in ovarian cancer with BRCA mutations and high Nmut. *BRCA1* methylation is associated with a significant decrease of BRCA1 transcript levels, higher levels of genome-wide LOH and, in this study, higher mutation burden [9,11,15]. Under selection of platinum treatment, it is possible *BRCA1* methylation may be reversible, and lead to the restoration of BRCA1 expression. In breast cancer xenografts, therapy resistant triple-negative cancer lost *BRCA1* promoter methylation and re-expressed the BRCA1 protein [17]. The epigenetic co-inactivation of other gene(s), for instance in pro-apoptotic pathway(s), is a possibility that could explain the worse outcome of patients with *BRCA1* methylation compared to those with *BRCA1* mutation. These possibilities remain open to future studies. 

Next-generation DNA sequencing is widely used in research and may become a clinical tool to detect specific and targetable mutations. A by-product of the increased use of whole-genome and whole exome sequencing studies may be a simple enumeration of genome-wide mutations, which may itself represent a genomic biomarker for predicting outcomes in patients receiving optimal surgery and chemotherapy. 

 Our observations are consistent with the concept that BRCA1 and BRCA2 regulate error-free repair of nucleotide damage and act to minimize single nucleotide mutations. There is evidence of BRCA1 DNA repair activity in pathways other than HR. Pathania, et al demonstrated BRCA1 played a role in repair of UV radiation-damaged DNA. BRCA1 participated in DNA replication-dependent but nucleotide excision repair (NER)-independent repair processes by promoting photoproduct excision and suppression of error-prone translesion synthesis (TLS) at UV-induced stalled replication forks [18]. High activity of TLS may be induced by FANCJ activity when there is loss of BRCA1 binding to FANCJ [19]. As TLS polymerases are error-prone, an up-regulation of TLS in the setting of BRCA1 deficiency may explain a higher rate of somatic nucleotide substitutions. 

 Whole genome sequencing in breast cancer identified a characteristic distribution of single nucleotide mutations with an increased overall mutation burden in both *BRCA1-* and *BRCA2*-associated tumors. All possible nucleotide substitutions were seen within 96 possible trinucleotide sequence contexts without predominant patterns of particular trinucleotides, which was a characteristic signature of both BRCA1- and *BRCA2*-associated breast cancers [14]. This characteristic appears consistent with loss of a key mechanism(s) for error-free DNA repair in addition to HR, or activation of an error-prone DNA replication process. 

 Other lines of evidence show differences between *BRCA1* and *BRCA2* mutation-associated ovarian cancers. These differences include relatively earlier onset in *BRCA1* than *BRCA2* germline mutation carriers, and a relatively better survival in patients with *BRCA2* than *BRCA1* mutation-associated tumors in comparison to that in patients with wtBRCA-associated ovarian cancer [6,15]. Our results show the same associations between tumor Nmut and treatment outcome in both BRCA1- and *BRCA2*-associated ovarian cancers. This observation is consistent with similar signatures of mutational processes in breast and ovarian cancers from patients with either *BRCA1* or *BRCA2* germline mutations [14,20]. There are other well-recognized similarities between BRCA1- and *BRCA2*-associated diseases. These similarities include HR-mediated DNA repair deficiencies, sensitivity to DNA damaging agents and PARP inhibitors, and reversion mutation-associated treatment resistance [3,6,7,9,21,22]. 

 A low mutation burden in tumors with either a homozygous *BRCA1* or *BRCA2* damaging mutation and LOH at the corresponding BRCA locus may be explained by activation of alternative mechanism(s) capable of bypassing the defect and restoring error-free DNA repair. Our knowledge of bypass pathways of repair is limited. Alternative activation of HR by concomitant loss of 53BP1 in BRCA1-deficient cells may restore resistance to PARP inhibitors, but does not change the sensitivity to cisplatin [23,24]. Reversion mutation of *BRCA1/2* genes in recurrent disease may result in resistance to platinum chemotherapy and PARP inhibitors, but is rarely found in the primary disease [21]. Our results are based on a relatively small set of patients carrying *BRCA1* and *BRCA2* mutations, and should be considered hypothesis generating until confirmed in a larger cohort. In addition, tumors may possess *de novo* mechanisms leading to resistance to chemotherapy and targeted treatments. Our preliminary results suggest low tumor Nmut may identify BRCA-associated primary tumors in which the original deficiency of BRCA1 and BRCA2 pathways, including impaired DNA repair, is compensated for by alternative pathways. 

## Materials and Methods

### Datasets

We obtained exome sequencing data of 316 high-grade serous ovarian cancers and follow-up information from TCGA [9]. Any sequence alteration in the ovarian tumor exome that was not present in the germline DNA sequence was called a somatic mutation and included both non-synonymous and synonymous changes. In the exome mutation data published by the TCGA consortium, a total of 19,356 somatic mutations were identified in the cohort, and most independently validated by a second assay using whole-genome amplification of a second sample from the same tumor [9]. Mutations that were not independently validated were computationally evaluated and had a high likelihood to be true mutations as described [9]. Based on TCGA mutation calls explained above, the total number of somatic mutations in the tumor exome (Nmut) was determined for each case (Table S1). Affymetrix SNP6 genotyping data and updated clinical information were obtained from the TCGA data portal (http://tcga-data.nci.nih.gov/tcga/, dbGaP accession no. phs000178.v5.p5, acquired 2011 Oct 27). *BRCA1* and *BRCA2* gene mutation status, *BRCA1* and *RAD51C* methylation status and ethnic/racial information were acquired from the cBIO SU2C data portal (http://cbio.mskcc.org/su2c-portal/). 

### Clinical assessment of therapy response

All patients underwent debulking surgery prior to platinum and taxane-based chemotherapy. The outcome of debulking surgery was the presence or absence of visible residual disease at the end of surgery; in TCGA the dimensions of residual disease were estimated. All patients received platinum-based chemotherapy after surgery. Chemotherapy resistance was defined as disease progression during first-line platinum-based chemotherapy or progression within 6 months after completion of first-line therapy [25]. Chemotherapy sensitivity was defined as progression-free survival longer than 6 months. 

### Bioinformatics analysis

Affymetrix SNP6 array data for tumor-normal pairs were normalized using the Aroma CRMAv2 algorithm, and B-allele fraction (BAF) was adjusted using the CalMaTe and TumorBoost Aroma packages[26-28]. Processed data were analyzed for LOH, allelic imbalance, copy number changes and normal cell contamination using ASCAT[29], as described elsewhere [12,29]. Nmut was determined by counting all mutation calls for each sample reported by the TCGA consortium (Table S1). Mutations include missense, nonsense, silent, frameshift and splice variants [9]. The median value for Nmut was determined for the cohorts and high Nmut was defined as those values above the median, and low Nmut was values equal to or below the median. Correlation was determined by the Spearman rank correlation coefficient. Statistical significance was assessed by the Wilcoxon rank-sum test for two-group comparison or by Kruskal-Wallis test for multiple-group comparison. Survival analysis was performed using Kaplan-Meier analysis and Cox regression. For Kaplan-Meier analysis, Nmut was dichotomized around its median value in study cohorts. In Cox regression, Nmut is continuous, but hazard ratio (HR) is reported per 10 mutations. The variables for multivariate analysis included Nmut, age, stage (II, III, IV), and residual disease (not visible, < 1 cm, 1-2 cm, and > 2 cm). All *P* values are 2-sided, and all bioinformatics analysis was performed in the R 2.15.2 statistical framework.

## Supporting Information

Table S1 **Genomic and ethnic/race information of TCGA ovarian cancer cohort used in the present study.**
(TXT)

Figure S1 **A) Total number of exome mutations (Nmut) in high-grade serous ovarian cancer carrying wtBRCA or mutated BRCA1/2 genes(**s**) (mBRCA).** The tumor Nmut is presented by dot plots. Median and 25-75 percentiles are indicated by horizontal lines. P-value is derived from Wilcoxon rank-sum test. **B**-**C**: Tumors were separated into Nmut high and low groups defined by the median Nmut across the whole cohort and compared to the rate of chemotherapy resistance. The significance of the differences was determined by Fisher’s exact test. OR: Odds Ratio. Confidence intervals in brackets. **B**) mBRCA, **C**) wtBRCA.
(TIF)

Figure S2 **Nmut and survival in mBRCA cases based on germline or somatic origin of the BRCA1/2 mutation.** **A**) Total number of exome mutations (Nmut) in high-grade serous ovarian cancer carrying mutated BRCA1/2 genes(s) of either germline or somatic origin. The tumor Nmut is presented by dot plots. Median and 25-75 percentiles are indicated by horizontal lines. P-value is derived from Wilcoxon rank-sum test. **B**) and **C**) Kaplan-Meier analysis comparing PFS (B) and OS (C) between serous ovarian cancer patients with either germline or somatic mBRCA.
(TIF)

Figure S3 **Position of mutations in BRCA1 and BRCA2 proteins by amino acid number, and their association with Nmut.** **A**) and **B**) shows BRCA1 (**A**) and BRCA2 (**B**), with the domains of BRCA1 and BRCA2 proteins illustrated with different colors on top of each panel. The Y-axis shows for each mBRCA tumor Nmut, with the corresponding position of the BRCA1/2 mutation indicated on the X-axis. Germline mutations are indicated in blue, somatic in red. Missense mutaitons are shown as diamonds. **C**) and **D**) shows Nmut by grouping the locations of BRCA mutiations according to relevant regions in BRCA1 and BRCA2, respectively. Red dotted lines on **A**) and **B**) shows the exact grouping cut-offs. P-values comparing Nmut by location is determined by a Kruskal-Wallis test.
(TIF)

Figure S4 **Nmut by BRCA1/2 mutations status, and by BRCA1 or RAD51C methylation status.** P-value is based on a Wilcoxon test, and compares each group to wtBRCA independently.
(TIF)

Figure S5 **Correlation of tumor Nmut with patient age at the time of diagnosis.** **A**) germline BRCA1/2 mutation carriers. **B**) germline or somatic BRCA1/2 mutations. **C**) wtBRCA tumors. Correlation between age and Nmut is determined by Spearman’s rank correlation coefficient.
(TIF)

Figure S6 **A) and B), correlation between tumor Nmut and the fraction of the genome with LOH (FLOH).** **C**) and **D**), correlation between tumor Nmut and the number of chromosome arms with telomeric allelic imbalance events (NtAI). BRCA genotype (mBRCA and wtBRCA) indicated above each panel.
(TIF)

Figure S7 **The influence of post-surgery residual disease on progression-free and overall survival in ovarian cancer using Kaplan-Meier analysis to compare patients with to patients without residual disease.** **A**) and **B**) mBRCA tumors. **C**) and **D**) wtBRCA tumors.
(TIF)

Figure S8 **Tumor Nmut and clinical treatment outcome in ovarian cancer patients with mBRCA tumors and residual disease or no residual disease.** **A**) and **B**) Kaplan-Meier analysis compared PFS and OS between high and low Nmut in ovarian cancer patients with mBRCA and no residual disease following debulking surgery. **C**) and **D**) PFS and OS between high and low Nmut in ovarian cancer patients with mBRCA and residual disease following debulking surgery. High and low Nmut is defined by median Nmut of all mBRCA cases.
(TIF)
