# Supplementary figures and images for: Tumor Mutation Burden Forecasts Outcome in Ovarian Cancer with BRCA1 or BRCA2 Mutations
Source: PLoS One. 2013 Nov 12;8(11):e80023. doi: 10.1371/journal.pone.0080023 (PMC3827141; doi:10.1371/journal.pone.0080023)

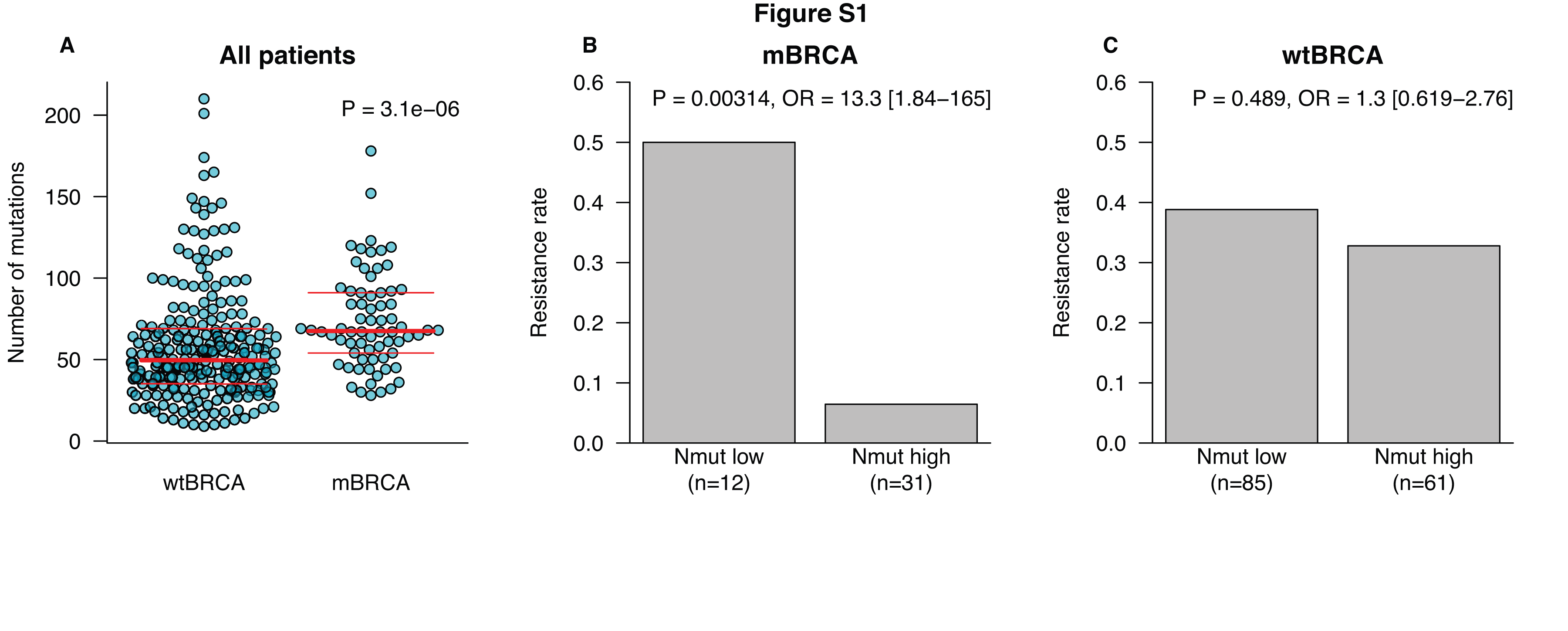

Supplement: Figure S1 — A) Total number of exome mutations (Nmut) in high-grade serous ovarian cancer carrying wtBRCA or mutated BRCA1/2 genes(s) (mBRCA). The tumor Nmut is presented by dot plots. Median and 25-75 percentiles are indicated by horizontal lines. P-value is derived from Wilcoxon rank-sum test. B-C: Tumors were separated into Nmut high and low groups defined by the median Nmut across the whole cohort and compared to the rate of chemotherapy resistance. The significance of the differences was determined by Fisher’s exact test. OR: Odds Ratio. Confidence intervals in brackets. B) mBRCA, C) wtBRCA. (TIF) [file pone.0080023.s002.tif]

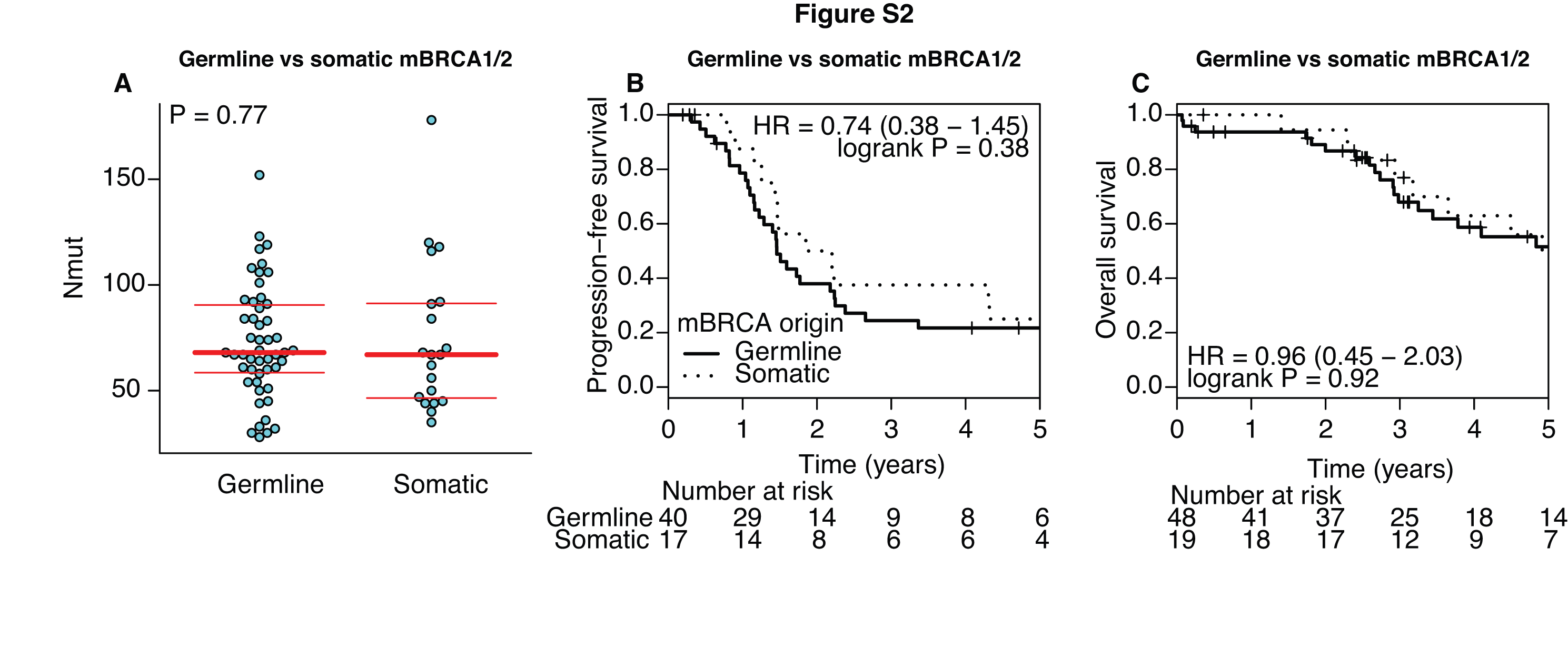

Supplement: Figure S2 — Nmut and survival in mBRCA cases based on germline or somatic origin of the BRCA1/2 mutation. A) Total number of exome mutations (Nmut) in high-grade serous ovarian cancer carrying mutated BRCA1/2 genes(s) of either germline or somatic origin. The tumor Nmut is presented by dot plots. Median and 25-75 percentiles are indicated by horizontal lines. P-value is derived from Wilcoxon rank-sum test. B) and C) Kaplan-Meier analysis comparing PFS (B) and OS (C) between serous ovarian cancer patients with either germline or somatic mBRCA. (TIF) [file pone.0080023.s003.tif]

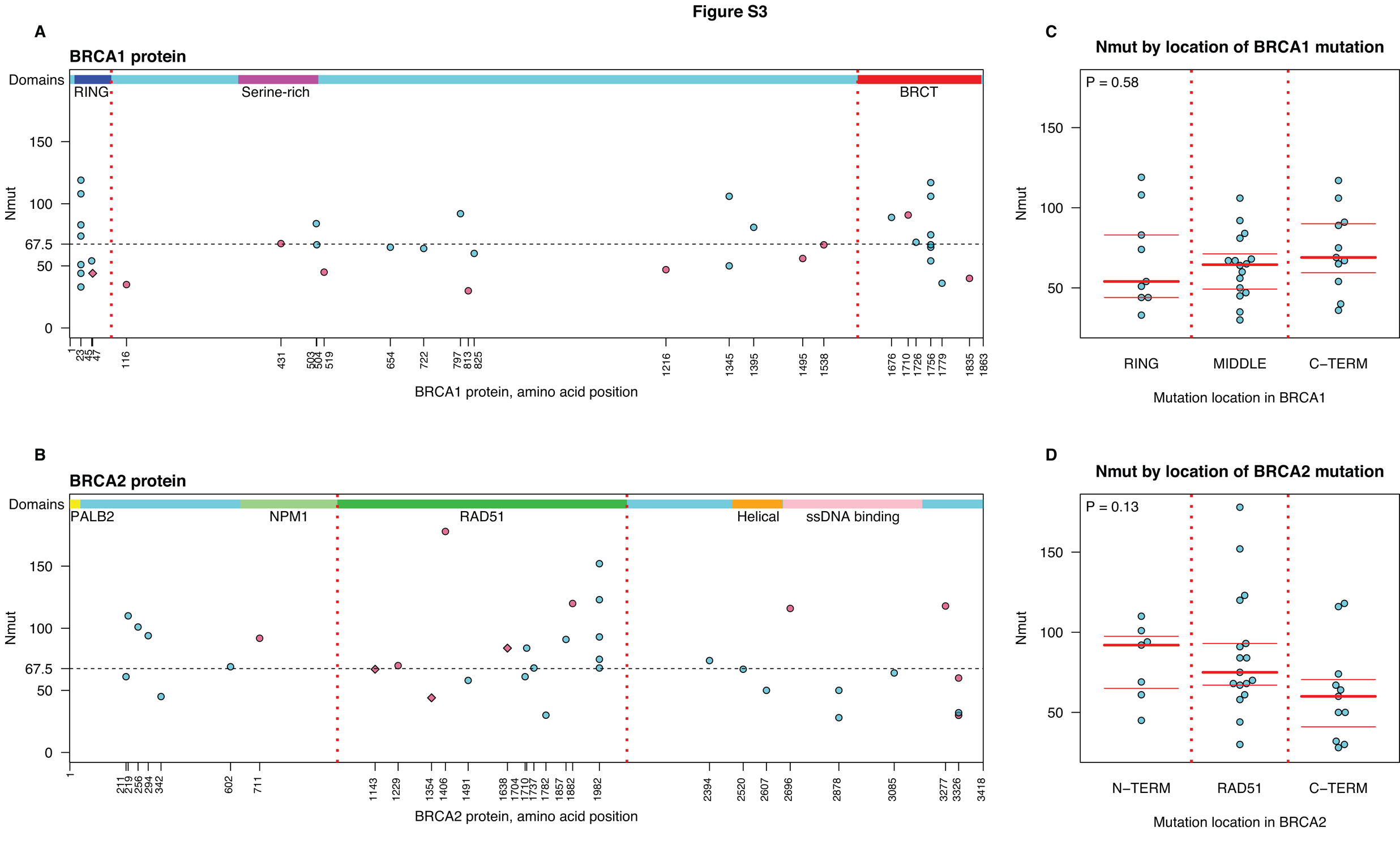

Supplement: Figure S3 — Position of mutations in BRCA1 and BRCA2 proteins by amino acid number, and their association with Nmut. A) and B) shows BRCA1 (A) and BRCA2 (B), with the domains of BRCA1 and BRCA2 proteins illustrated with different colors on top of each panel. The Y-axis shows for each mBRCA tumor Nmut, with the corresponding position of the BRCA1/2 mutation indicated on the X-axis. Germline mutations are indicated in blue, somatic in red. Missense mutaitons are shown as diamonds. C) and D) shows Nmut by grouping the locations of BRCA mutiations according to relevant regions in BRCA1 and BRCA2, respectively. Red dotted lines on A) and B) shows the exact grouping cut-offs. P-values comparing Nmut by location is determined by a Kruskal-Wallis test. (TIF) [file pone.0080023.s004.tif]

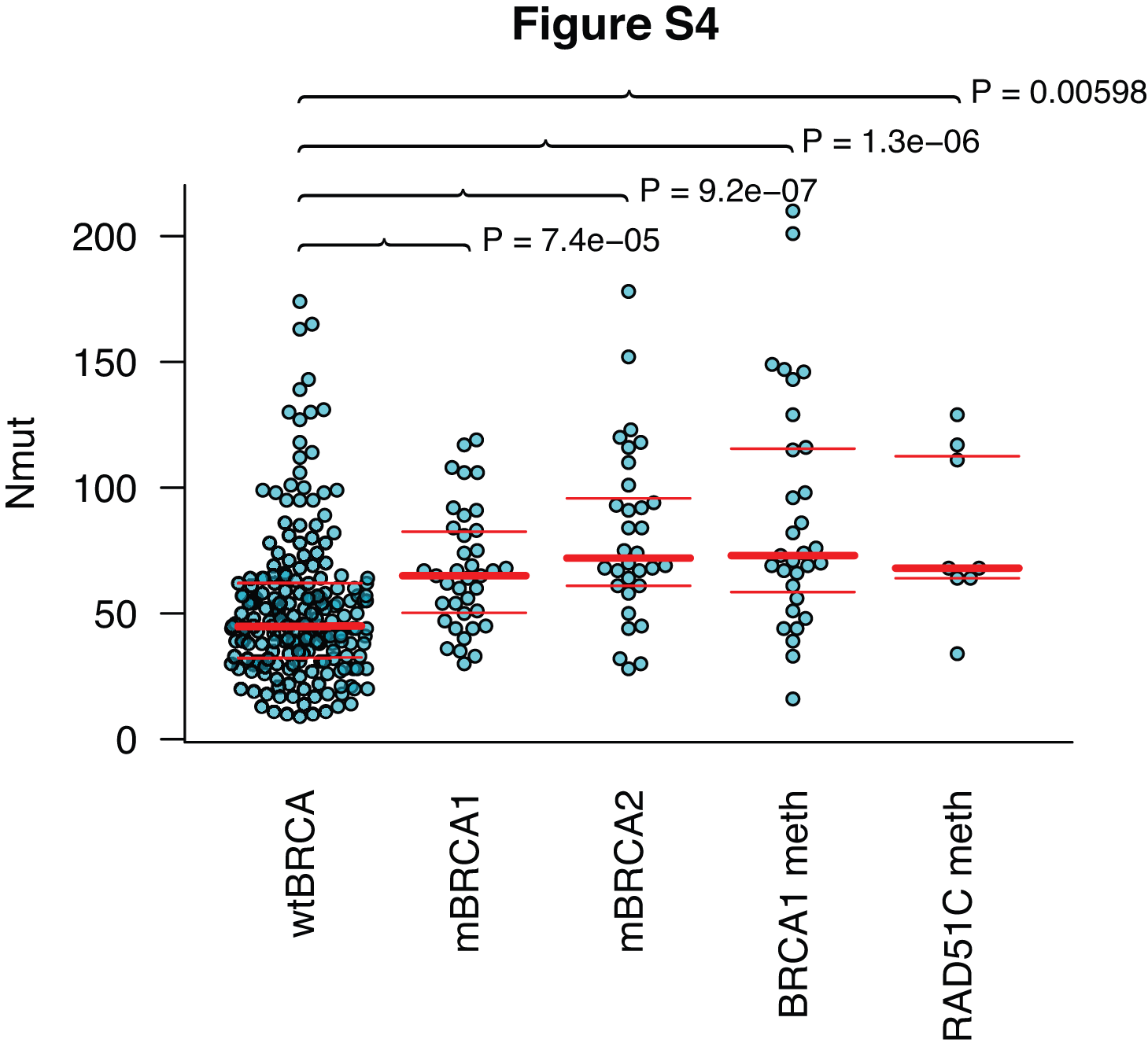

Supplement: Figure S4 — Nmut by BRCA1/2 mutations status, and by BRCA1 or RAD51C methylation status. P-value is based on a Wilcoxon test, and compares each group to wtBRCA independently. (TIF) [file pone.0080023.s005.tif]

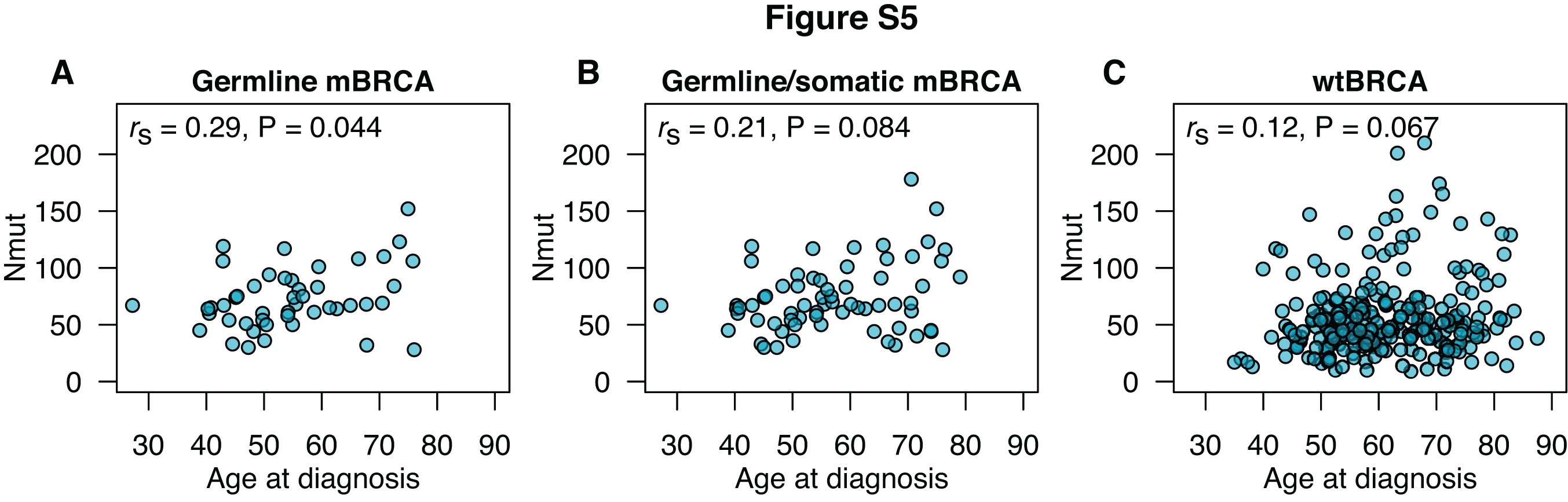

Supplement: Figure S5 — Correlation of tumor Nmut with patient age at the time of diagnosis. A) germline BRCA1/2 mutation carriers. B) germline or somatic BRCA1/2 mutations. C) wtBRCA tumors. Correlation between age and Nmut is determined by Spearman’s rank correlation coefficient. (TIF) [file pone.0080023.s006.tif]

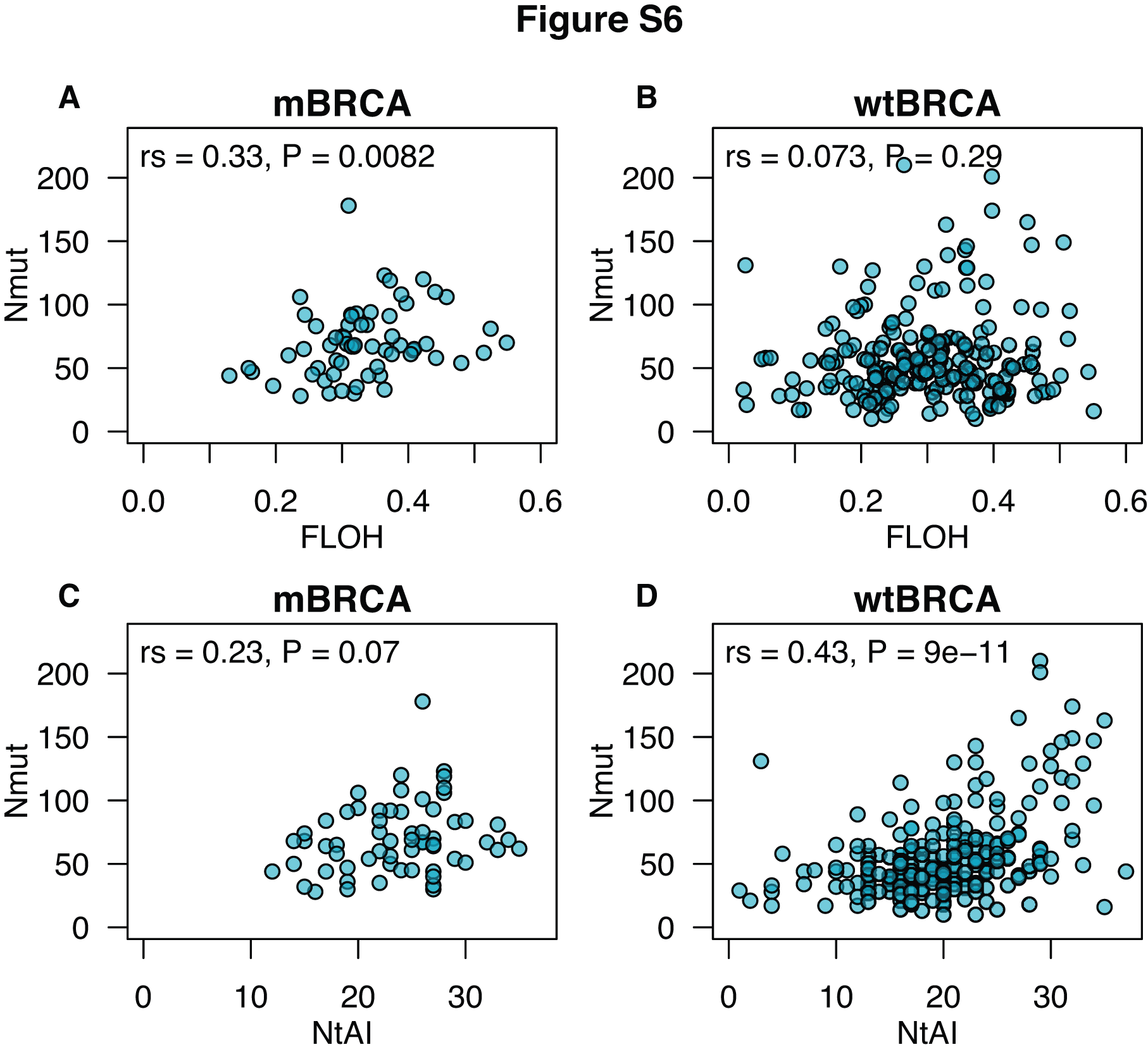

Supplement: Figure S6 — A) and B), correlation between tumor Nmut and the fraction of the genome with LOH (FLOH). C) and D), correlation between tumor Nmut and the number of chromosome arms with telomeric allelic imbalance events (NtAI). BRCA genotype (mBRCA and wtBRCA) indicated above each panel. (TIF) [file pone.0080023.s007.tif]

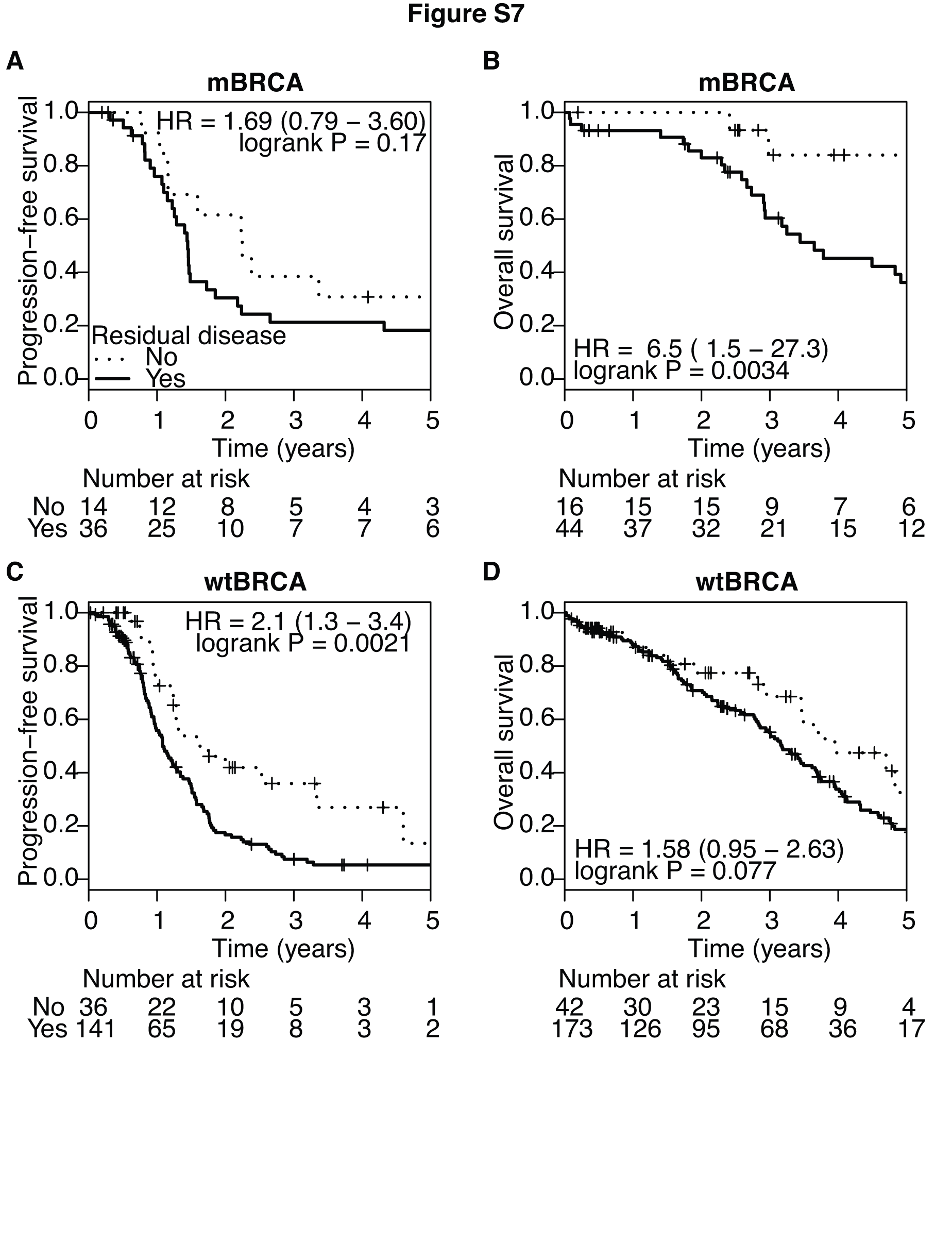

Supplement: Figure S7 — The influence of post-surgery residual disease on progression-free and overall survival in ovarian cancer using Kaplan-Meier analysis to compare patients with to patients without residual disease. A) and B) mBRCA tumors. C) and D) wtBRCA tumors. (TIF) [file pone.0080023.s008.tif]

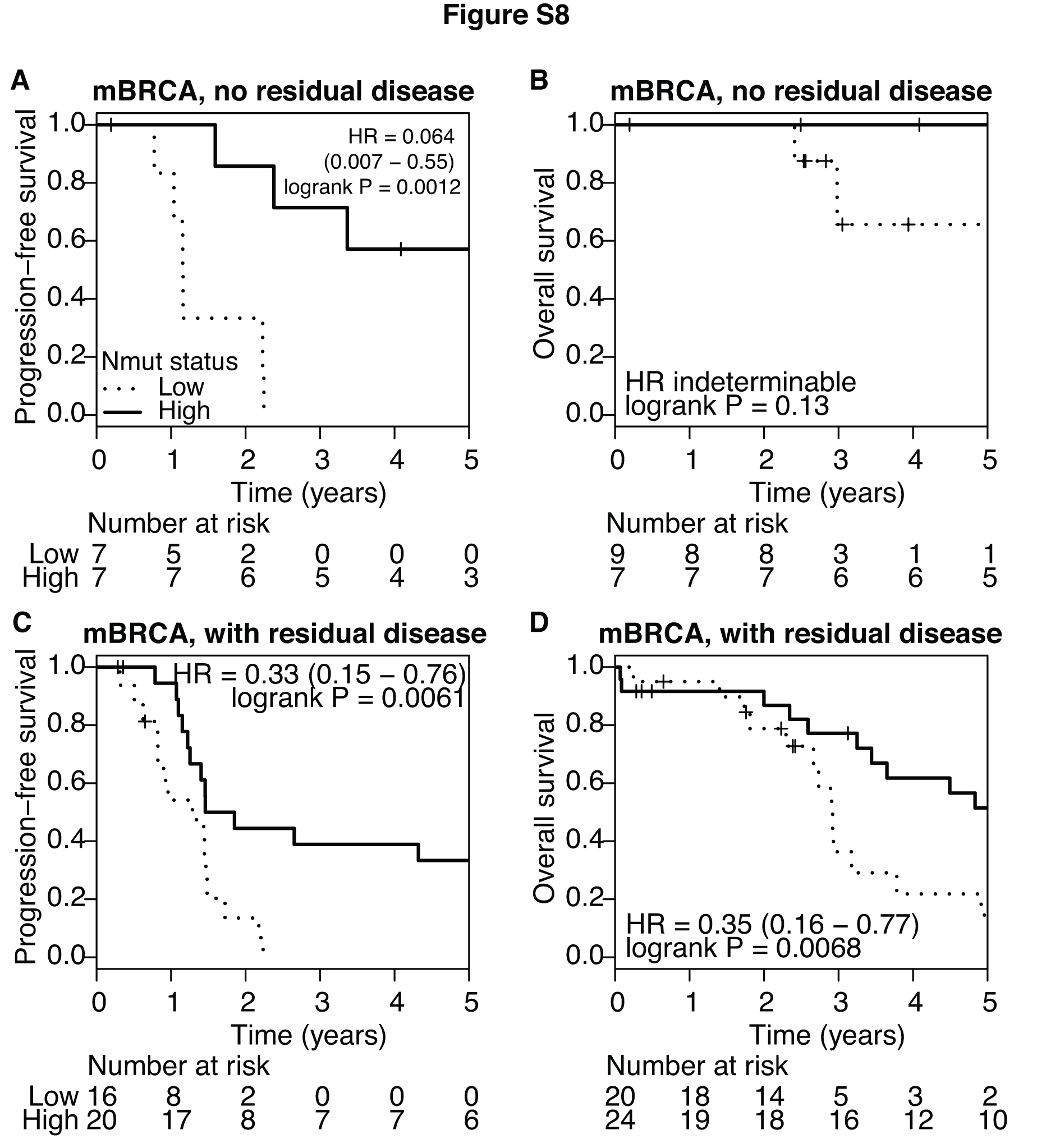

Supplement: Figure S8 — Tumor Nmut and clinical treatment outcome in ovarian cancer patients with mBRCA tumors and residual disease or no residual disease. A) and B) Kaplan-Meier analysis compared PFS and OS between high and low Nmut in ovarian cancer patients with mBRCA and no residual disease following debulking surgery. C) and D) PFS and OS between high and low Nmut in ovarian cancer patients with mBRCA and residual disease following debulking surgery. High and low Nmut is defined by median Nmut of all mBRCA cases. (TIF) [file pone.0080023.s009.tif]
